# Effect of microfluidic channel integration onto gold microelectrode on its redox electrochemistry

**DOI:** 10.55730/1300-0527.3532

**Published:** 2022-12-07

**Authors:** Ahmet UÇAR, Zahraa ALI HAJOOL, Hamed GHORBANPOOR, Reza DİDARİAN, Fatma DOĞAN GÜZEL

**Affiliations:** 1 Department of Energy Systems Engineering, Faculty of Engineering and Natural Sciences, Ankara Yildirim Beyazit University, Ankara, Turkey; 2 Department of Metallurgical and Materials Engineering, Faculty of Engineering and Natural Sciences, Ankara Yildirim Beyazit University, Ankara, Turkey; 3 Department of Biomedical Engineering, Faculty of Engineering and Architecture, Eskisehir Osmangazi University, Eskisehir, Turkey; 4 Department of Biomedical Engineering, Faculty of Engineering and Natural Sciences, Ankara Yildirim Beyazit University, Ankara, Turkey; 5 Department of Mechanical Engineering, Faculty of Engineering and Natural Sciences, Ankara Yildirim Beyazit University, Ankara, Turkey

**Keywords:** Microfluidics, lab-on-chip, gold, microelectrode, macroelectrode, redox electrochemistry, sensing, cyclic voltammetry

## Abstract

Microfluidic systems have attracted significant interest in recent years as they are extensively employed in lab-on-chip and organ-on-chip research. Their combination with electrochemical platforms offers many advantages, promising a high potential for sensing applications, still the microfluidic-channel integration onto electrodes might induce challenges related to changes in signal-to-noise ratios and mass transport conditions. In this study, we investigated the effect of microfluidic channel integration in redox behavior of thermally deposited gold thin film microelectrodes by voltammetric (CV and SWV) electrochemical measurements. Using different dimensions of PDMS microfluidic channels (i.e. widths of 50, 100, 250, and 500 μm) and a constant electrode dimension (200 μm), we analyzed the relationship between altered electroactive area and electrochemical response against target redox molecules. The increases in electroactive area which were determined by the microfluidic channel sizes were in well-correlation with the obtained CV and SWV redox currents as expected. There was no significant decrease in signal-to-noise ratio in microchannel-integrated electrodes. AFM and SEM characterization demonstrated that thermally deposited thin film electrodes had significantly lower (approximately 25 fold) surface roughness in comparison to commercial screen-printed electrodes. Additionally, we have observed a clear microelectrode-to-macroelectrode transition, from hemispherical to linear (planar) diffusion in other terms, with the increasing channel size.

## 1. Introduction

Microfluidic systems have been employed in many lab-on-a-chip [1–3] and organ-on-a-chip [4,5] applications in the last decade. They still draw significant attention especially in medical diagnosis and treatment due to their advantages such as rapid analysis, durability, ease of use, high sensitivity and accuracy, reduced sample consumption, applicability for miniaturization and low fabrication costs [6,7]. Although they can be used as stand-alone in labeling [8], isolation/capturing [9], and optical sensing [10], they are also combined with electrochemical systems. The integration of microfluidic systems with electrodes can result in the development of electrochemical sensors/biosensors with enhanced capabilities including but not limited to higher sensitivity and selectivity against target analytes, multiplexed detection using arrays of electrodes and translation to point-of-care testing [11].

The size (dimensions) of an electrode is a very important parameter that could alter its electrochemical response [12]. Microelectrodes having one critical dimension of a micrometer scale are known to show several differences such as lower currents, iR drop, and capacitances but enhanced mass transport, current densities and signal-to-noise ratios over larger electrodes (e.g., macroelectrodes which are larger than 100 μm). Mass transport can be defined as the transportation of chemical molecules from the bulk solution to the electrode surface and this occurs in three different modes: i) diffusion, ii) migration, and iii) convection. Assuming these chemical molecules are redox-active, enhancement in mass transport can be shown as a leading factor for increased electroanalytical capabilities of microelectrodes compared to macroelectrodes. The first reason of the enhancement in mass transport for microelectrodes is the rapid evolution of the steady-state diffusion regime [13]. While the linear diffusion is effective for macroelectrodes due to larger diffusion field thickness than electrode size, this is not the case for electrodes having smaller sizes (microelectrodes and nanoelectrodes) where diffusion field is comparable with electrode dimensions, resulting in the generation of hemispherical diffusion. Another reason is the minimized natural convection in electrodes with smaller sizes. Although convection on the surface reduces the electrode current densities, this becomes more negligible for microelectrodes which are dominated by increased diffusion effects [14]. However, it should be noted that reducing electrode dimension can bring in several disadvantages such as lower currents recorded, potential evaporation of liquid probe/sample during preparation and/or analysis and the risk of inaccessible analyte due to geometrical restrictions [15]. All of these issues can be avoided by fabricating the microelectrodes in array architectures [16,17]. Electrode arrays also create an opportunity for multianalyte detection.

Microelectrode fabrication has improved over time due to the growth of semiconductor industry and the corresponding development in new photolithographic methods that allow the production of reproducible electrodes with well-defined shapes and dimensions [18]. Although the most common microelectrodes are metal disc electrodes produced by encapsulating the metal wire into an insulator material such as glass or plastic, they are available in various geometries including cones, bands, hemispheres, and cylinders [19]. In recent times, it has also become very common to produce microelectrodes as thin films deposited onto various substrates in desired dimensions and shapes using shadow masks [20], photolithography [21], or maskless techniques [22]. The areas where microelectrodes are employed include classical sensors/biosensors [23], microfluidic sensors [3,24,25], chromatographic devices [26], and scanning electrochemical microscopy (SECM) [27].

The devices which have been developed by the integration of microfluidic systems and electrodes are being widely used in sensing applications; therefore, this is not a new field. However, there is really a lack of comprehensive analysis which exhibits the changes in electroanalytical performance of the microfluidic-integrated electrodes in comparison to nonintegrated (off-chip) electrodes. We have recently reported on the effect of microchannel-microelectrode integration on electrochemical impedance spectroscopy (EIS) measurements and changes in recorded electrochemical noise [28]. It was seen that microelectrodes combined with microchannels having a width of 1000 μm showed the best response in terms of lower noise levels in EIS measurements in comparison to narrower channels used. However, we emphasized that further analyses should be conducted for a better understanding of the effects of microelectrode-microchannel integration. Here in this work, we investigate the effect of microfluidic channel integration on the redox behavior of microelectrodes using voltammetric techniques, cyclic voltammetry (CV) and square wave voltammetry (SWV). To achieve this, we placed four different microchannels with varying widths (50, 150, 250, and 500 μm) on gold microelectrodes deposited onto the glass substrates via thermal evaporation. The electrode system was based on two electrodes consisting of a working electrode (WE) and a reference/counter electrode (RE-CE) with widths of 200 μm and 1 mm, respectively. We have compared the results with responses from off-chip electrodes and commercial screen-printed electrodes (SPEs). In addition to electrochemical measurements, we also performed morphological characterizations in order to understand any physical reasons for changes in electrochemical behaviors. We believe that the findings of this work will present a clearer picture of the improvements/limitations of microchannel-microelectrode combinations, which would eventually enhance the production of integrated devices.

## 2. Materials and methods

### 2.1. Chemicals

Potassium ferri/ferrocyanide (FFC), sulfuric acid, ethanol, isopropanol, acetone, 10X phosphate-buffered saline (PBS) were purchased from Sigma Aldrich, diluted when required and used as received otherwise. All reagents were of analytical grade and all solutions were prepared using deionized water (DI).

### 2.2. Electrode design, production, and characterization

Electrode was previously designed using AutoCAD software. The design was based on depositing onto a 26 mm × 26 mm glass slide, having a working electrode (WE), a reference/counter electrode (RE-CE) with widths of 200 μm and 1 mm respectively, and 4 mm-wide contact pads. Shadow mask was produced by Photo Lab (Cambridge, UK). For electrode production, thermal evaporation was used to deposit metal layers onto a glass substrate (UNAM, Bilkent University, Turkey). The glass slides were cleaned in an ultrasonic bath for 15 min with isopropanol and acetone, before they were dried with nitrogen gas. Firstly, 100 nm of chromium (Cr) (99.99% Nanovak, Turkey) was first deposited to enhance the adhesion of the gold layer to the glass surface. Then, 300 nm of gold (Au) (99.99%) (Nanovak, Turkey) was deposited onto this adhesion layer. Preliminary characterization of the deposited electrodes was carried out using an optical microscope (Euromex, Arnhem, The Netherlands). Atomic force microscopy (AFM) (Quesant Q-Scope 250, Euromex, Netherlands) was used for the morphological characterization of the electrode surface. To do this, the surface was cleaned, rinsed with deionized water for 10 s, dried with nitrogen gas, and AFM measurements were performed by using the following parameters (scan rate: 1 Hz, scan angle: 0, resolution: 512, lateral resolution: 1 nm). Scanning electron microscopy (SEM, HITACHI SU5000 FE-SEM instrument) was also used to further analyze the surface morphology of the deposited gold electrodes (Central Laboratory of Ankara Yıldırım Beyazıt University, Turkey). The SEM imaging parameters were as follows: size: 800 × 600, pixel size: 13229.17, working distance: 63519.59 μm, emission current: 125,000 nA. The thickness and width profile of the deposited layers were characterized by a Dektak Profiler (Bruker Dektak, UNAM, Bilkent University, Turkey).

### 2.3. Microfluidic channel design, fabrication, and chip assembly

The chip used for microfluidic channel fabrication was designed using AutoCAD software and an acetate photomask was produced by Çözüm Tanıtım (Ankara, Turkey). Depth of the microchannels was 50 μm. Four different microchannels were applied with widths of 50, 150, 250, and 500 μm. Production of the silicon template was carried out using the traditional optical photolithography method. Then, approximately 3 mL of photoresist SU-8 2050 (Laurell Technologies Corporation, USA) was applied on a 3-inch silicon wafer. The wafer was uniformly spin-coated with the photoresist. This was followed by several steps including soft baking, UV exposure, postbaking, development process (using propylene glycol methyl ether acetate, PGMEA), and hard baking. The microfluidic chips were produced using the soft lithography process using polydimethylsiloxane (PDMS). The PDMS kit (Sylgard 184, Dow Corning USA) contains two components: silicon base (prepolymer) and curing agent (cross-linker). Silicon template was placed in a petri dish, then the PDMS basic elastomer mixture (silicon base and curing agent in a ratio of 10:1) and a well curing agent were left at 50 °C for 24 h. Assembly of the integrated chip was performed using a custom-made plasma device. Beforehand, the microelectrodes deposited onto glass slides and PDMS chips were first cleaned in 70% ethanol in an ultrasound bath for 5 min, then rinsed with deionized water and dried with nitrogen gas. The PDMS was then chemically bonded to the electrode-deposited glass slides through plasma exposure for 25 s. It was further treated at 50 °C on the hotplate for 2 h before applying electrically conductive adhesive silver epoxy (M.G. Chemicals, Ontario, Canada) on contact pads (for electrode connections to the potentiostat) and final curing at 50 °C on the hotplate for 24 h.

### 2.4. Electrochemical measurements

Electrochemical measurements were both performed with (on-chip) and without (off-chip) the integration of PDMS microchannels. Off-chip experiments were carried out using two types of electrodes. The first was commercially obtained screen-printed gold electrodes (220AT (WE 4 mm) and C223AT (WE 1.6 mm), Metrohm, Switzerland) and labeled as SPE in the text. The second one was a PDMS layer as a large hole on a gold electrode-deposited glass slide and labeled as off-chip ME in the text. On-chip experiments were performed using electrodes integrated with microfluidic channels having varying channel widths (50 μm, 150 μm, 250 μm, 500 μm) and labeled in the text referring to their channel size (i.e. ME-50). Before any use, the electrodes were first rinsed with DI for 10 s, then dried and electrochemically cleaned with a solution of 0.1 M H_2_SO_4_. In on-chip experiments, DI water and H_2_SO_4_ solution were injected into the microchannels by using a syringe pump. In order to ease the connection and stabilization of the integrated devices, a simple device holder was first designed and fabricated, and used during electrochemical measurements. The electrical connection was provided via aluminum needles on holder clamps and conductive silver epoxy paste on contact pads. Cyclic voltammetry (CV) was applied between 0 and +1.6 V at a scan rate of 0.1 V s^−^^1^ using a potentiostat device (PalmSens PS4, the Netherlands) in order to electrochemically clean the electrodes. The number of scans taken was accordingly arranged to achieve a stable and characteristic voltammogram for gold electrode. Following the electrode cleaning, 1 mM potassium ferri/ferrocyanide in 1X PBS solution was used as a redox mediator, and CV and SWV measurements were performed between −0.6 V and +0.6 V at a scan rate of 0.1 V s^−^^1^. Ag was used as the reference electrode for SPEs, while gold was used as both counter and reference electrode for integrated devices. PSTrace v5.8 software was used to perform electrochemical measurements and all data were analyzed and plotted using OriginLab 2019b.

## 3. Results and discussion

### 3.1. Integration of microfluidic channels and microelectrodes

**Figure 1:**
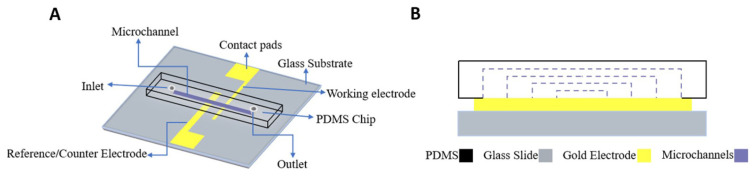
(A) Device schematic and (B) cross-sectional view of the microchannel integrated on-chip gold electrodes. Dotted lines represent varying widths of the channel.

**Figure 2:**
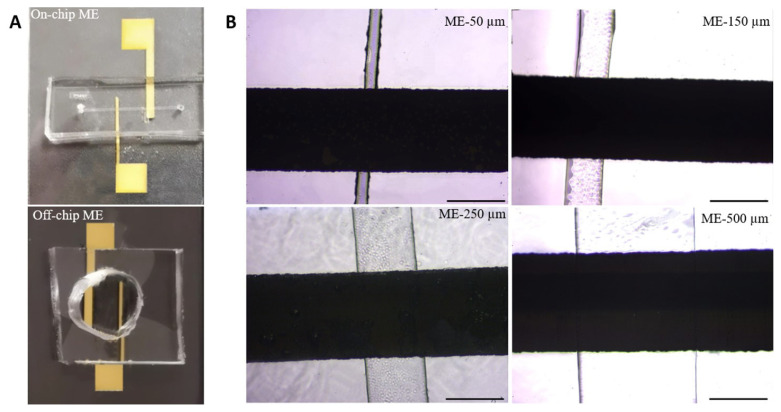
(A) Images of on-chip (microchannel integrated) and off-chip microelectrodes, (B) optical microscope images of on-chip microelectrodes with varying microfluidic channel sizes of 50, 150, 250, and 500 μm.

In order to achieve microfluidic channel integration on microelectrodes and to investigate any effect in their electrochemical responses, different fabrication and characterization tools/techniques were employed in this study. The electrode design included two-electrodes which consisted of a working electrode (WE) and a reference/counter electrode (RE-CE) with widths of 200 μm and 1 mm, respectively. They were fabricated as gold thin films (with a thickness of approximately 300 nm) on cleaned glass substrates by thermal evaporation technique using an aluminum shadow mask for patterning. To increase the adhesion between deposited gold layers and glass substrates, a thin chromium layer (approximately 100 nm) was first deposited. Photolithography and soft lithography techniques were used for the production of microfluidic channels. Following the mask design and silicon template production, polydimethylsiloxane (PDMS)-based microfluidic channels were prepared in four different channel widths of 50, 150, 250, and 500 μm, which were finally bonded by oxygen plasma treatment to glass slides which were patterned with gold thin film electrodes. The integration was easy and reproducible as long as the surfaces are precleaned sufficiently. The profilometer measurements (not presented here) showed that the obtained electrode and channel dimensions were close to the projected values. The microfluidic channel-integrated electrodes are named as on-chip electrodes throughout the text, while other types of electrodes used for comparison, are named as off-chip (gold thin film electrodes which were not channel-integrated) and SPEs. Figure 1 shows the device schematic and cross-sectional view of the microchannel integrated on-chip gold electrodes. The dashed blue lines in the cross-sectional view correspond to the integrated channel widths, resulting in the defined electroactive area for electrodes. Photography images of on-chip (microchannel integrated) and off-chip microelectrodes, and optical microscope images of on-chip microelectrodes with varying integrated microfluidic channel widths of 50, 150, 250, and 500 μm are shown in Figure 2. As seen, off-chip microelectrodes were not bonded with a fluidic channel and instead it was only combined with an aperture-opened PDMS layer to define a fixed active electrode area and to avoid any solution leakage. The presented optical microscope images revealed the successful and reproducible microelectrode and microchannel fabrication and integration in terms of the projected dimensions and defined borders. These images were also used for calculation of the geometrical electroactive area for the devices.

### 3.2. Morphological characterization

**Figure 3:**
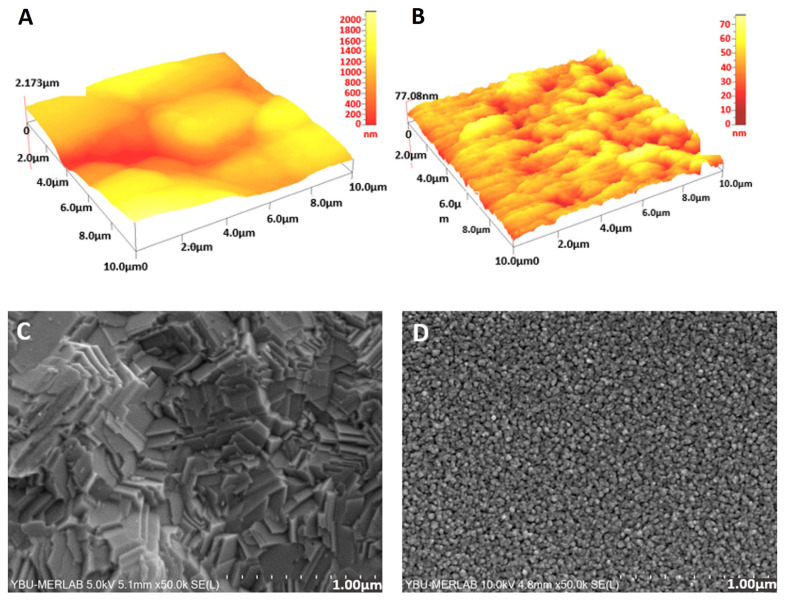
3D AFM images of (A) screen printed and (B) deposited thin film gold microelectrode surfaces and SEM surface microstructure images of (C) screen printed and (D) deposited thin film gold microelectrodes.

In addition to profilometer measurements performed to check the reproducibility of electrode and channel production, atomic force microscopy (AFM) and scanning electron microscopy (SEM) were used for morphological assessment of gold thin film microelectrode surfaces fabricated via thermal evaporation technique. Figures 3A and 3B illustrate 3D AFM images of screen-printed and thermally evaporated thin film gold electrode surfaces. As clearly noticed, the surface roughness of the fabricated thin film electrodes was significantly less (approximately 25 fold) than screen-printed electrodes. SEM findings (Figures 3C and 3D) were also consistent, showing a lower roughness and more organized microstructure for thin film electrodes. This was quite reasonable when considering the difference between two types of electrodes in terms of the electrode material and deposition technique employed, still there cannot be a conclusion here that a rougher or smoother surface is better, because it should be noted that it completely depends on a specific application. For instance, it was reported that macroscopic roughness (on the order of hundreds of nanometers) had only influence on the capacitance, while microscopic roughness (up to tens of nanometers) influenced both the layer ideality and the capacitance for electrodes coated with self-assembled monolayers (SAMs) [29]. Moreover, a recent study has shown that the relationship between surface roughness and hydrophobicity was totally irrelevant even for different samples of the same material [30].

### 3.3. Electrochemical response

**Figure 4:**
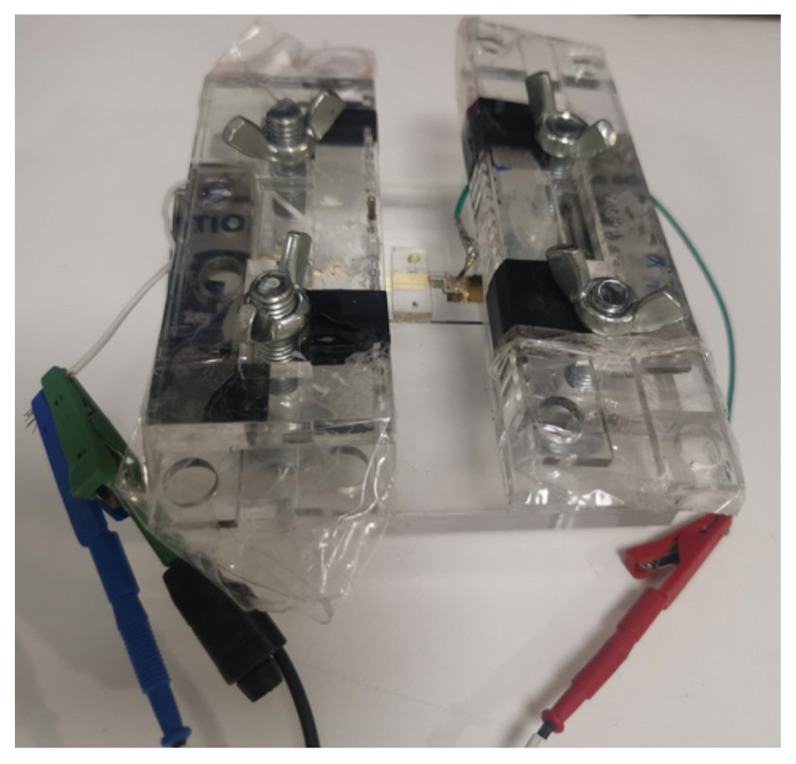
Image of the holder used for stabilization and connection of the integrated devices for electrochemical measurements.

Electrodes can be combined with micro- [31] and nanofluidic [32] channels before employment in many different applications due to advantages of miniaturization. The most significant advantage is the enhanced sensitivities in lower sample volumes and concentrations [25]. However, both miniaturization and microfluidic integration can become a challenge which could lead to electrochemical limitations such as low currents, increased noise levels and electrode connection difficulties [33]. Therefore, systematic studies are required to show the effects of miniaturization and microfluidic integration in electrochemical behavior of electrodes. As a result of this requirement, we here investigated the effect of microfluidic channel size (varying widths) on reduction-oxidation (redox) responses of microelectrodes integrated with microfluidic channels. To achieve this, voltammetric measurements were recorded using cyclic voltammetry (CV) and square wave voltammetry (SWV) techniques on on-chip, off-chip, and screen-printed electrodes. In order to stabilize the chip-based electrodes and to connect them to the potentiostat for electrochemical measurements, a simplistic device holder (Figure 4) consisting of aluminum needles was specifically designed, fabricated, and utilized during the experiments.

**Figure 5:**
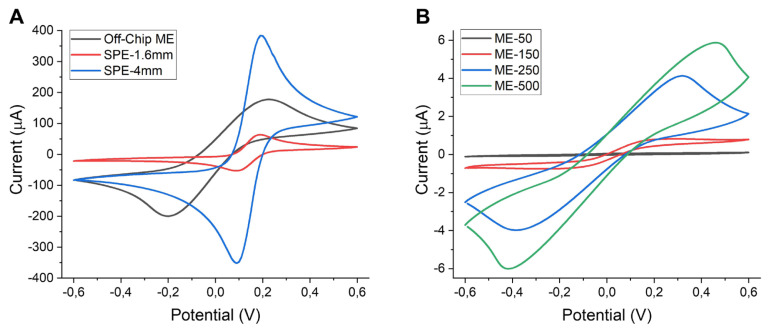
CVs of (A) nonintegrated (off-chip and screen printed) and (B) microchannel-integrated on-chip gold electrodes in 1 mM potassium ferri/ferrocyanide in 1X PBS. CVs were recorded between potentials of −0.6 and 0.6 V at a scan rate of 100 mV s^−^^1^

**Figure 6:**
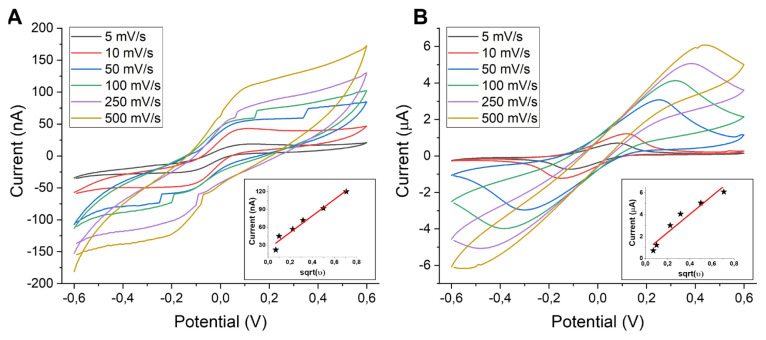
CVs of on-chip (A) ME-50 μm and (B) ME-250 μm recorded between potentials of −0.6 and 0.6 V at varying scan rates (5, 10, 50, 100, 250, 500 mV s^−^^1^) in 1 mM potassium ferri/ferrocyanide in 1X PBS. Insets show the average of oxidation and reduction peak currents plotted against the square root of scan rates.

**Figure 7:**
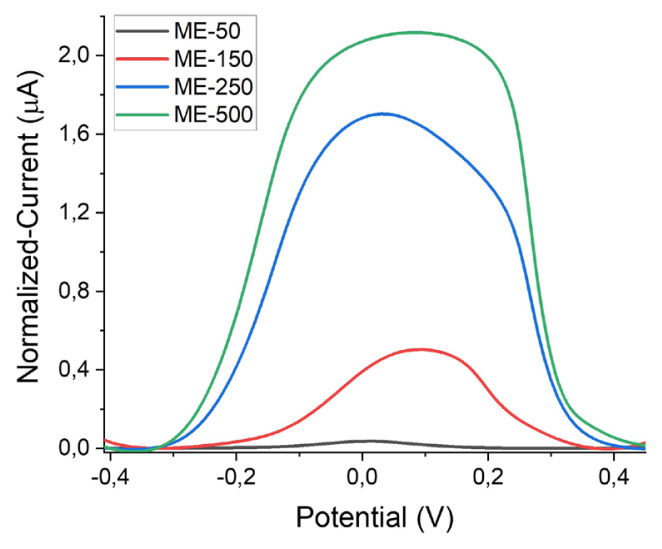
SWVs (background-subtracted) of microchannel-integrated on-chip gold microelectrodes in 1 mM potassium ferri/ferrocyanide in 1X PBS. SWVs were recorded between potentials of −0.6 and 0.6 V at a scan rate of 100 mV s^−^^1^

All the electrodes were first electrochemically cleaned using the CV method in sulfuric acid where a voltage range was applied between 0 and +1.6 V at a scan rate of 0.1 V s^−^^1^. Electrochemical characterization of a clean electrode is generally performed using a widely used redox agent, which is repeatedly reduced and oxidized at an electrode surface. Figure 5 shows the cyclic voltammograms of non-integrated (off-chip and screen printed) and microchannel-integrated on-chip gold electrodes recorded in 1 mM potassium ferri/ferrocyanide solution between −0.6 and 0.6 V at a scan rate of 100 mV s^−^^1^. The voltage range represented in the figure was chosen in accordance with the redox chemistry of ferri/ferrocyanide. In both nonintegrated and integrated electrodes, a clearly seen expected correlation exists between the current and electrode area. Considering the electroactive area of off-chip electrodes is somewhere between the areas of two types of SPEs, which have 1.6- and 4-mm WE diameters, the obtained oxidation peak height (approximately 180 μA) was found to be consistent. A clear shift can be seen in reduction peak potential of the off-chip electrode in comparison to SPE voltammograms. However, this was also expected because Ag was used as a pseudo reference electrode for SPEs, while gold was used as both counter and reference electrode for integrated devices. For on-chip electrode voltammograms, the currents were significantly lower (<6 μA) as the active electrode areas were also smaller due to being limited by integrated microchannel widths. It is worth noting that the differences in the shapes of the recorded CVs for on-chip electrodes were significant. Due to having different mass transport (hemispherical versus linear diffusion) onto electrode surface, a sigmoidal shape is usually obtained for electrodes with smaller dimensions such as micro- or nanoelectrodes, whereas peaks are characteristic for macroelectrodes [13]. This was also found to be valid for our case where the voltammogram shapes of ME-50 and ME-150 were sigmoidal, while the peaks were obtained for ME-250 and ME-500. This was most probably due to the change of mass transport regime based on increased microchannel size. To check if there is any dependence of the obtained current amounts and voltammogram shapes on CV scan rate, ME-50 and ME-250 were selected as candidates and cycled at varying scan rates of 5, 10, 50, 100, 250, 500 mV s^−^^1^ in the same solution which includes potassium ferri/ferrocyanide redox molecules, as presented in Figure 6. Again, mostly sigmoidal shapes were seen for ME-50 while there were peaks instead for ME-250. The real expectation was that both electrodes would show peaks at faster scan rates due to the promoted linear diffusion because there would be no time for steady-state equilibrium [34]. However, this was not clearly seen in our results. This may have been due to the measurement parameters used or a different surface-related redox phenomenon; therefore, there is a need for further investigation about this. Additionally, the registered oxidation peak currents were plotted against the square root of scan rates (Figure 6 insets). The linear correlation between the obtained current and scan rate confirmed that the redox process was chemically reversible at both electrode surfaces, as fundamentally suggested by Randles–Ševčík equation [35]. In electroanalysis, square wave voltammetry (SWV) is usually preferred over linear sweep methods like CV due to being a more sensitive technique minimizing the capacitive currents [36]. Therefore, we have also performed SWV measurements for microchannel-integrated electrodes, as represented in Figure 7. Similar to CV results, the obtained peak currents were increasing with fluidic channel width due to corresponding increases in electrode area. The voltammograms recorded for ME-250 and ME-500 were broader than expected, which might be due to either parameter optimization or electrode capacitance, which needs further assessment for better understanding. Gamby and coworkers [37] reported that a similar platform based on a microfluidic-integrated two-electrode system had a better DNA sensing performance than conventional electrodes, which resulted from the forced convection and decreased diffusion layer thickness leading to faster target collection toward the biosensor. This exhibits the potential of a similar design for enhanced (bio)sensing performance. In another study, interdigitated Au gold microelectrode arrays were fabricated and employed for the detection of 4-aminophenol where the sensitivity was found to be increased (with reported LoD of 0.1 nM) due to sample confinement and controlled flow in microfluidic-integrated electrodes [38]. Basheer and coworkers [39] investigated the effect of the microfluidic channel size on the sensor performance using CV technique. In this work, the channels with varying widths (100, 400, 700, and 1000 μm) were fabricated and integrated onto the electrodes (with widths of 100 μm and 200 μm). They also observed that the electrokinetic properties were depending on both parameters; the microchannel and the electrode size. Table represents the related studies of microfluidic channel-integrated electrodes, their electrochemical characterization methods and significant remarks for comparison.

## 4. Conclusion

Herein we investigated the changes in electrochemical response of gold thin film electrodes following the integration with PDMS-based microfluidic channels with varying widths. To achieve this, gold electrodes were patterned as thin films on glass substrates by thermal evaporation. PDMS microchannels with varying widths were produced using optical- and soft lithography techniques. Varying widths when bonded onto WE of microelectrodes enabled us to change the effective area of the electrodes. AFM and SEM characterization showed that substantially lower surface roughness was obtained compared to commercially available screen-printed electrodes. Electrochemical measurements confirmed that the integration of microchannels and corresponding changes in electroactive area was effective in redox processes. Moreover, the effect of the channel width was found to be significant to alter mass transport conditions at the electrode surface, where the larger channel size promoted the linear (planar) diffusion. We believe that the findings set the basics for discussions on electrochemical fundamentals in relation of microchannel integration onto microelectrodes. On the other hand, the approach to minimize the microelectrode size via microfluidic integration also offers ease and low-cost in terms of fabrication of smaller scale microelectrodes in comparison to the production by relatively expensive semiconductor technology. Further studies are required to assess the (bio)sensing performance of the platform.

## Figures and Tables

**Table:** List of recent studies on microfluidic channel-integrated electrodes structures.

Electrode type	WE dimension	Channel width	Technique	Significant remarks	Ref
Deposited Au film	30 μm	300 μm	CV	Limit of detection of DNA hybridization increased in the presence of flow	[37]
Sputtered Au film	10 μm	200 μm	Amperometry	Sensor sensitivity against 4-aminophenol increased in the presence of flow	[38]
Not known	100 & 200 μm	100–1000 μm	CV	Highest sensitivity reported for the microchannel size of 700 μm and electrode size of 200 μm	[39]
Thermally deposited Au	100 μm	150–1000 μm	CV, EIS	Higher noises reported for EIS & CV measurements for the integrated channels with widths narrower than 1000μm	[28]
Thermally deposited Au	200 μm	50–500 μm	CV, SWV	Hemispherical to linear diffusion observed with the increasing channel size (electroactive area)	This study
